# The importance of time in nutrient regulation: a case study with spotted-wing Drosophila (*Drosophila suzukii*)

**DOI:** 10.3389/finsc.2023.1105531

**Published:** 2023-07-20

**Authors:** Carrie Deans, William Hutchison

**Affiliations:** Department of Entomology, University of Minnesota, St. Paul, MN, United States

**Keywords:** geometric framework, invasive species, intake target, nutrition, fruit fly, macronutrients

## Abstract

**Introduction:**

The ability of living organisms to acquire the nutrients needed to carry out required physiological functions has important consequences for fitness. However, an organism must not simply meet the requirements for individual nutrients, but must ingest an optimal balance of multiple nutrients. Despite this, animals rarely consume truly balanced resources, and instead commonly feed selectively across multiple unbalanced resources to reach an optimal balance, i.e., intake target. Nutritional research has predominantly focused on the behavioral strategies employed during nutrient regulation, as well as the fitness consequence of failing to meet intake targets, but little work has been done on the temporal aspects of this process. For instance, within what timeframe must organisms reach their intake target before a fitness cost is incurred? Hours, days, weeks?

**Methods:**

In this study, we investigated how nutrient regulation interval impacts consumption and performance in adult female spotted-wing Drosophila (Drosophila suzukii). Females were constrained to either a protein- orcarbohydrate-biased diet over different time intervals and at different schedules, while control flies were constrained to one diet for the entire feeding period.

**Results:**

Regulation interval had a significant impact on feeding behavior and consumption. Total consumption was highest on the shorter interval treatments, where diets were alternated more frequently, and declined as the interval period increased. The relative consumption of both diets was statistically-different across intervals and was higher for the carbohydrate-biased diet. Consumption of the protein-biased diet was more variable across intervals and was more strongly impacted by the daily timing of diet switches. Performance data showed that shorter regulation intervals led to longer fly lifespans, a result commonly observed in studies exploring the impacts of diet macronutrient ratio variability on performance.

**Discussion:**

These results show that the temporal aspects of nutrition, such as feeding intervals and the timing of resource availability, can have strong impacts on feeding behavior, nutrient regulation, and fitness. These results provide an insight into how consumers may deal with changes in host phenology, the availability of hosts, and changes in nutrient availability within hosts. Understanding these mechanisms will be important for predicting responses to changes in nutrient cycling and resource availability mediated by natural and anthropogenic habitat modifications, such as global climate change.

## 1 Introduction

The geometric framework of nutrition (GF) has been an incredibly useful tool for understanding the behavioral, ecological, and physiological aspects of nutrition (1–4). While the GF can be used to determine the fitness effect of any combination of dietary compounds, including nutrients, allelochemicals, water, etc., macronutrients have a particularly important effect on animal fitness (5–9). Specifically, the balance of dietary protein (p) and carbohydrates (c) have primary effects on survival and reproduction across taxa. GF studies have also shown that different nutritional optima, i.e., known as intake targets and denoted by a specific p:c ratio, exist for meeting specific physiological demands, such as maximizing growth, reproduction, and longevity, or contending with immunological challenges and other stressors (8, 10–14). Understanding these nutritional relationships have allowed researchers to identify the strategies employed by consumers to meet their nutritional demands, as well as the consequences of failing to do so.

To mitigate discrepancies between a desired intake target and an available resource(s), consumers can regulate their macronutrient intake pre-ingestively by altering their feeding behavior. When a consumer has access to multiple resources, it can selectively feed on each to systematically acquire the desired balance (12, 15–19). However, when a consumer only has access to one resource that is not in-line with its intake target, it can employ different strategies to mitigate nutritional imbalances. It can either ingest enough of the resource to reach the requirement for one macronutrient, while either over- or under-ingesting the other, or it can feed to an intermediate point that minimizes excesses and deficits (15, 17). Across nutritional options, animals exhibit an array of different behaviors and physiological processes that help them deal with nutritional imbalances, including learning (17, 20, 21), compensatory feeding (22, 23), alterations in digestive efficiencies (22, 24), and dietary restriction (11, 25). The majority of GF studies have focused on these strategies as they relate to mismatches in macronutrient ratio and/or total macronutrient content. Very few studies, however, have explored the temporal aspects of macronutrient regulation. For example, over what timeframe must an intake target be reached? How much time do consumers have to mitigate nutritional imbalances without incurring additional physiological costs (days, weeks, months)? How do consumers deal with the uncertainty of fluctuations in resources availability over time? Studies focused on these types of questions are lacking among nutritional studies, despite a strong ecological justification for them. Just as macronutrient profiles vary across resource types, they also vary over time. Daily, seasonal, and/or yearly fluctuations in resource quantity and quality are common across different types of resources. The spatio-temporal variability in plant macronutrient profiles has been well-documented (14, 18, 26, 27), as well as its impacts on insect nutrient regulation and performance (12, 14, 18, 27–29), yet the temporal aspects of this variability remain poorly studied.

It certainly seems intuitive that the longer it takes a consumer to reach its nutritional requirements, the longer physiological processes may be delayed. This suggests that optimal timeframes for regulation exist and may be process-specific. On the other hand, there may be some lability in response time if resources can be re-allocated from other less time-sensitive processes to fuel higher-priority functions. Understanding the impacts of these scenarios will ultimately require a better understanding of regulation over different timeframes. In this study, we allowed adult female flies to feed on two unbalanced diets, one at a time, over different time intervals, to explore the impact that regulation time had on fly fitness and their ability to reach an intake target. In nature, access to different resources vary over time, which can affect the interval over which organisms regulate their nutrient intake. This is particularly true for fungivores, like fruit flies, that are impacted by, not only by phenological changes in plant nutrients, but also the microbial colonization of plant tissues (19, 30). Understanding the temporal aspects of nutrient regulation will not only improve our understanding of insect nutritional ecology but will also be integral to further understanding the mechanisms that consumers will use to deal with the impacts of global climate change on patterns of nutrient cycling and host availability.

## 2 Methods

### 2.1 Fly culture

The flies used in these experiments came from a lab colony established in 2018 from infested raspberry samples collected from the University of Minnesota’s UMORE Station near Rosemount, MN. The culture was reared on a standard cornmeal-based oligidic diet (cornmeal, sugar, agar, nutritional yeast, propionic acid, methyl paraben, ethanol) in narrow polystyrene vials (Genesee Scientific Corporation) (31). Flies were transferred to new diet every 2-3 days. Colonies and experimental flies were kept in a walk-in chamber at ambient lab temperature, which ranged from 20-22°C, under a 14:10 light-dark cycle.

### 2.2 Artificial diets

The artificial diet described in Lihoreau et al. (32) was to used create the adult treatment diets, with slight alterations. Our diets consisted of a 1:1 ratio of whey and casein, sucrose, and nutritional yeast as the primary macronutrient sources. Vanderzant vitamin mixture provided micronutrients, while propionic acid and methylparaben were added as anti-microbials. All ingredients were set in 2% agar. The amounts of yeast, Vanderzant mix, and anti-microbials were standardized across diets, while the relative proportions of whey/casein and sucrose were adjusted to produce three diets with the same total macronutrient content (45g/L) but different protein-to-carbohydrate (P:C) ratios. We formulated a diet with a P:C ratio that matched the self-selected intake target of *D. suzukii*, 1:3 (19, 33), one with a carbohydrate-biased ratio relative to the intake target, 1:9, and one with a protein-biased ratio relative to the intake target, 3:1. All three diet ratios fall within the range of macronutrient profiles found for different fruit hosts utilized by SWD, as described by Young et al. (34) and Deans and Hutchison (19).

### 2.3 Treatments

Our diet treatments consisted of three controls, where flies were kept on a 1:3, 1:9, or 3:1 diet throughout the entire experiment, and six diet interval treatments, where flies had access to either a 1:9 or 3:1 p:c diet at different intervals over the course of a 16-hr feeding period. The feeding period ran from 8 am to 12 am, and flies only had access to water (in the form of 2% agar gel) for the rest of the day, from 12am to 8 am. The 16-hour feeding period was divided into six different intervals, defined as a period of time where flies had an equal amount of time to feed on both diets. This included an 8-hr interval, where diets were switched every 4 hr, two 16-hr intervals, where diets were switched every 8 hr, either at 12 am (16a) or 4 pm (16b), one 32-hr interval, where diets were switched each day, a 48-hr interval, where diets were switched every 1.5 days, and a 64-hr interval where, diets were switched every 2 days. **Table 1** shows the time schedule associated with each interval treatment. The name of each treatment describes the interval of time that the flies were exposed to both diets and thus, the entire interval over which they were allowed to regulate their intake across both. For example, in the 8-hr treatment, diets were switched every 4 hours so flies were exposed to both diets over an 8-hour interval, which occurred twice daily. In the 32-hour treatment, flies were exposed to one diet for an entire 16-hr feeding period and the second diet for another 16-hr interval, which occurred over the course of 2 days. The 16a and 16b treatments had the same interval but different switching time throughout the 16-hr feeding period. Because *D. suzukii* are crepuscular (35–37), these treatments allowed us to determine if the timing of diet switching impacted feeding behavior in addition to the interval.

**Table 1:** Diet schedule for each interval treatment across the 16-hour feeding period for the consumption experiment.

Treatment	Day	8am -12 pm	12pm – 4 pm	4 pm- 8 pm	8 pm- 12 am
8	1				
	2				
	3				
	4				
	5				
	6				
16a	1				
	2				
	3				
	4				
	5				
	6				
16b	1				
	2				
	3				
	4				
	5				
	6				
32	1				
	2				
	3				
	4				
	5				
	6				
48	1				
	2				
	3				
	4				
	5				
	6				
64	1				
	2				
	3				
	4				
	5				
	6				

The performance experiment maintained the same schedule but over 8 days. The starting diet was alternated across replicates within the interval treatments to avoid any confounding effects.

The gray and white blocks of time show the availability of different diets and the transition between colors shows at what time intervals the diets were switched.

### 2.4 Experimental protocol

Two experiments were done using the treatment diets. The first, determined how consumption varied across treatment intervals. The second, explored the impact of the treatment intervals on fly survival and performance. For the consumption experiment, 8 newly-eclosed (within 24 hours) female flies were placed into clear plastic vials containing their respective treatment diets (N=4). Flies were switched to different diets according to their treatment schedule. The flies were temporarily removed and each vial was weighed at the beginning and end of the day (16-hr period), as well as the total number of alive flies, so that consumption ((starting mass-ending mass)/number of flies) could be calculated. This experiment was carried out for 6 days and the data were used to determine if consumption of each diet varied across controls and interval treatments.

For the performance experiment, newly-eclosed (within 24 hours) female flies were moved into clear plastic vials (under CO2 anaesthetization) containing their respective treatment diets (N=16). They were housed individually with two male flies from the lab colony, which were introduced into each vial 24 hours after the females for mating. These mating flies were present throughout the experiment and replaced as needed to ensure than two males were always available. Diet switching was carried out at the treatment schedule for 8 days, after which female and mating flies were placed on an optimal 1:3 diet (19, 33) and allowed to feed and mate throughout their remaining life cycle. Flies were placed on new diet as needed. Mortality was recorded daily to determine fly lifespan and the total number of pupae and eclosing F1 flies were also recorded for each female.

### 2.5 Data analysis

Consumption of each diet was measured and used to calculate the average protein and carbohydrate intake, as well as the p:c ratio of consumed diet, for each treatment. An ANOVA was used to determine differences in total diet consumption and p:c intake between across the interval and control treatments. Separate generalized linear models were also done for each interval treatment to look at consumption over time. Each interval treatment was analyzed separately because consumption was measured over a different number of days depending on the treatment. For the 8-, 16a-, and 16b-hr treatments, a diet x time model was used, since both diets were administered within each day. For the 32-, 48-, 64-hr, and control treatments, a total consumption x time model was used to determine differences diet consumption over time because only one diet was available per day. Data were log-transformed when necessary to meet normality assumptions. A MANOVA was used to determine differences in the relative consumption of the 1:9 and 3:1 diets across interval treatments. An ANOVA was also used to determine differences in fly performance, including lifespan, pupal rate and eclosion success, across controls and interval treatments. These data were rank-transformed to meet normality assumptions. A Tukey HSD test was used for all *post-hoc* comparisons when significant effects were found, except for datasets with unequal variances in which case a Dunnett T3 *post hoc* test was used. All statistics were performed using SPSS v.27 (38).

## 3 Results

### 3.1 Total consumption

The amount of time that flies had to regulate their intake of the 1:9 and 3:1 diets had a significant impact on total diet consumption. **Table 2** shows that there were significant differences in total diet consumption across interval treatments and controls (P < 0.0001). In general, consumption was highest on the shorter-interval treatments (8-, 16a-, and 16b-hr) and declined as the interval period increased (**Table 3**). Flies in the shorter-interval diets ingested approximately 1.5 times as much diet as those in the longer-interval treatments and twice as much as those in the control treatments (**Table 3**).

**Table 2:** ANOVA results for average diet consumption across all interval and control treatments, as well as the intake p:c ratio of consumed diet across the interval treatments (including the controls).

Dependent Variable	Factor	df	F Stat	P-Value
total consumption	treatment	8	11.406	**<0.0001**
p:c ratio	treatment	5	8.206	**<0.0001**

Bolded values indicate statistically significant P-values (P ≤ 0.05). Consumption and p:c ratio were log-transformed to meet normality assumptions.

**Table 3:** Average total diet consumption across the interval treatments and controls (± SE).

Treatment	Consumption	
8	0.2800	± 0.0204
16a	0.2294	± 0.0140
16b	0.2110	± 0.0200
32	0.1216	± 0.0081
48	0.1874	± 0.0325
64	0.1454	± 0.0114
1:3	0.1240	± 0.0056
1:9	0.1223	± 0.0079
3:1	0.1112	± 0.0126

### 3.2 Relative consumption and nutrient regulation

**Figure 1:**
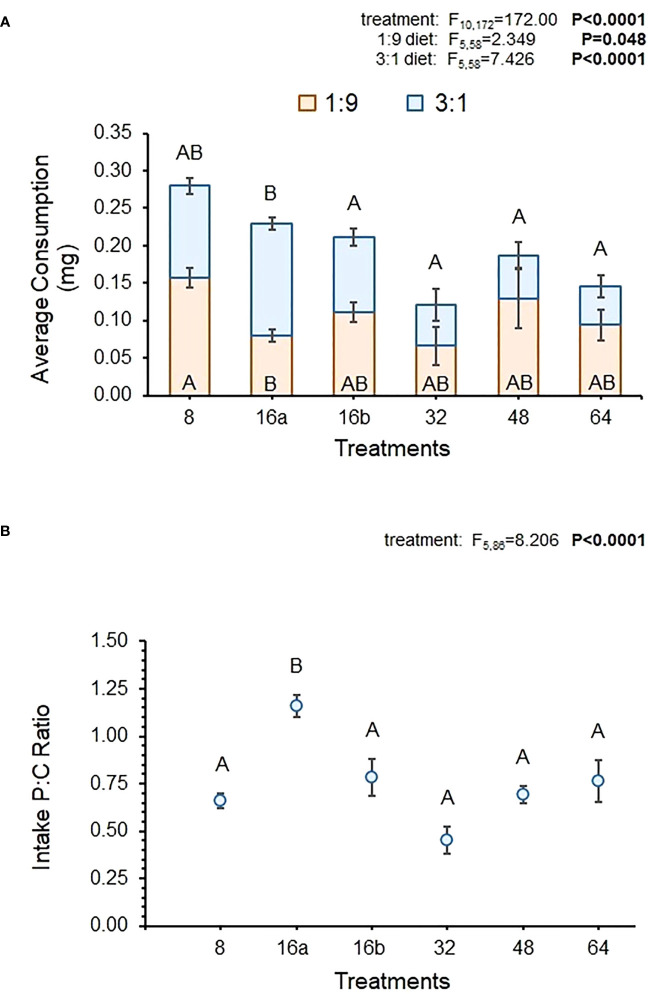
Average total consumption for each diet across all dates **(A)** and the average p:c ratio of consumed diet across all dates **(B)** in the interval treatments. MANOVA statistics (Pillai’s Trace) for diet consumption across the interval treatments (not including the controls) and the subsequent univariate statistics are shown for each diet. Bolded values indicate statistically significant P-values (P ≤ 0.05). Different letters indicate significant *post-hoc* (Tukey HSD) differences between diets and/or interval treatments (P ≤ 0.05).

The MANOVA results showed that over the entire feeding period, relative consumption of the 1:9 and 3:1 diets did vary across interval treatments (**Figure 1A**). The univariate results indicated that consumption of both diets was statistically-different across interval treatments but the consumption of the carbohydrate-biased 1:9 diet was 1.2 times higher than the protein-rich 3:1 diet. **Figure 1A** shows that consumption of the 1:9 diet was similar across treatments, with only differences being apparent between the 16a-hr and 8-hr treatment, where consumption was significantly higher in the 8-hr treatment (**Table 4**). There were no differences in the consumption of the 1:9 diet between the 16a- and 16b-hr treatments. Consumption of the 3:1 diet was more variable, being the highest in the 16a-hr treatment and lowest in the 64-hr treatment. Consumption was also higher in the 16a-hr compared to the 16b-hr treatment (**Table 4**). In general, flies in the shorter-interval treatments ate more of the 3:1 diet and those in the longer-interval treatments ate more of the 1:9 diet (**Figure 1A**).

**Table 4:** P-values for the MANOVA *post-hoc* analysis (Dunnett T3) for the consumption of the 1:9 and 3:1 diets across interval treatments.

Diet	Treatment	8	16a	16b	32	48	64
1:9	8	–	**0.001**	0.211	0.088	0.252	0.187
	16a	**0.001**	–	0.661	1.00	0.996	1.00
	16b	0.211	0.661	–	0.852	1.00	1.00
	32	0.088	1.00	0.852	–	0.987	0.996
	48	0.252	0.996	1.00	0.987	–	1.00
	64	0.187	1.00	1.00	0.996	1.00	
3:1	8	–	0.426	0.996	0.264	0.197	0.056
	16a	0.426	–	**0.019**	**0.015**	**0.001**	**<0.0001**
	16b	0.996	**0.019**	–	0.606	0.600	0.233
	32	0.264	**0.015**	0.606	–	1.00	1.00
	48	0.197	**0.001**	0.600	1.00	–	1.00
	64	0.056	**<0.0001**	0.233	1.00	1.00	–

Bolded values indicate statistically-significant P-values (P ≤ 0.05).

Despite differences in diet-specific consumption, the average p:c ratio of fly intake over the course of the experiment did not vary much across treatments. There was a statistically-significant effect of treatment on intake p:c, but **Figure 1B** shows that all treatments had similar ratio except for the 16a-hr treatment. Average p:c ratio varied from 0.45 to 1.16, with all treatments except 16a-hr exhibiting ratios below 1. Surprisingly, variability in intake ratio was not greater in the longer-interval treatments, despite flies in these treatments having fewer opportunities to regulate their intake over the course of the experiment (**Figure 1B**).

### 3.3 Consumption over time

**Figure 2:**
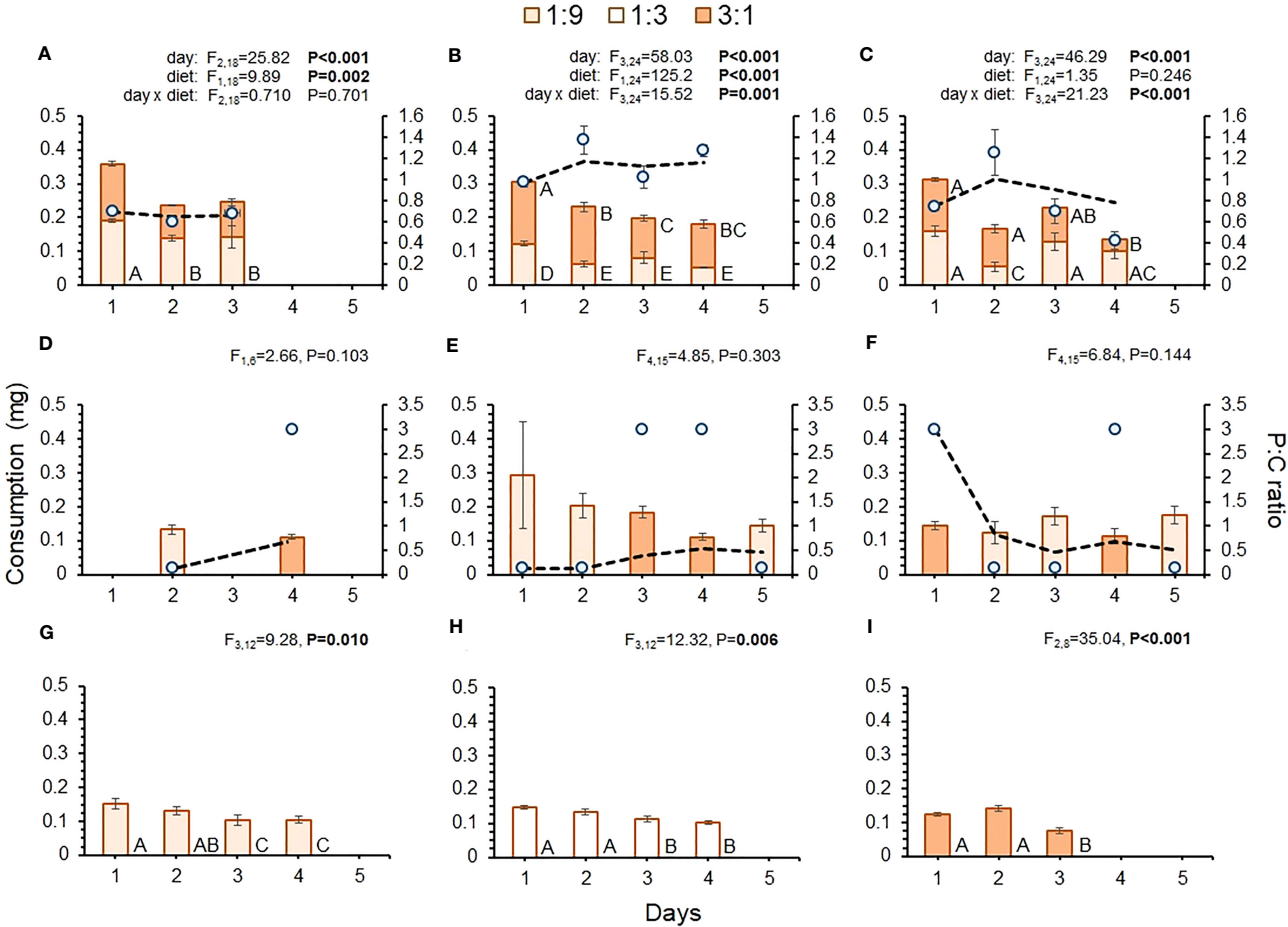
Consumption of each diet by day (bars) for each treatment: **(A)** 8-hr, **(B)** 16a-hr, **(C)** 16b-hr, **(D)** 32-hr, **(E)** 48-hr, **(F)** 64-hr, **(G)** 1:9 control, **(H)** 1:3 control, and **(I)** 3:1 control. Blue dots show the average p:c ratio of intake per day, while the dashed black line shows the average p:c ratio of cumulative intake across days. Generalized linear models were performed for consumption across the interval treatments where both diets were present each day and for the interval treatments where only one diet was available per day. Different letters indicate significant *post-hoc* differences between diets and/or time point for each diet (P ≤ 0.05).

There were considerable differences in daily consumption across interval treatments (**Figures 2A-F**). The 8-, 16a-, and 16b-hr treatments allowed flies to feed on both diets within a 24-hr period, while the 32-, 48-, 64-hr treatments only allowed flies to feed on one diet per day(s). **Figures 2A-F** shows that there was much more variability in total daily consumption across the shorter-interval treatments, as indicated by significant day or day-by-diet interactions for these treatments. Across all treatments, including the controls, there was a general trend of high initial daily consumption followed by reduced daily consumption as time passed. There was also considerable variability in the relative daily consumption of diets across interval treatments. Flies in the 8-hr treatment consumed significantly more of the 1:9 than the 3:1 diet on days 2 and 3 (**Figure 2A**), while those in the 16a-hr treatment consumed more of the 3:1 diet each day (**Figure 2B**). Flies in the 16b-hr treatment exhibited variable consumption of each diet across days (**Figure 2C**), with consumption of the 1:9 diet being greater on day 2, lower on day 4, and similar to the 3:1 diet on days 1 and 3. While trends of variable diet consumption across the different diets were apparent, there were no significant day effects in the longer-interval treatments, indicating that consumption of diet was similar over time, regardless of diet p:c ratio (**Figures 2D, E**). Despite only having access to one diet, there were significant differences in daily consumption in all control treatment, with consumptions being high initially and lessening over time (**Figures 2G-I**).

While flies in the shorter-interval treatments had the opportunity to regulate their nutrient intake within a 24-hr period, those in the longer-interval treatments did not. As such, the p:c ratio of daily intake was set by the diet that was available each day. As a result, the daily intake ratio was much more variable for flies in the longer-interval treatments than the shorter-interval treatments (**Figures 2A-F**). In the longer-interval treatments, intake ratios were initially high or low, depending on the diet they were offered at the beginning of the experiment, but over time these treatments ended up having cumulative p:c intakes that were rather similar to the shorter-interval treatments (**Figures 2D-F**).

### 3.4 Fly performance

**Figure 3:**
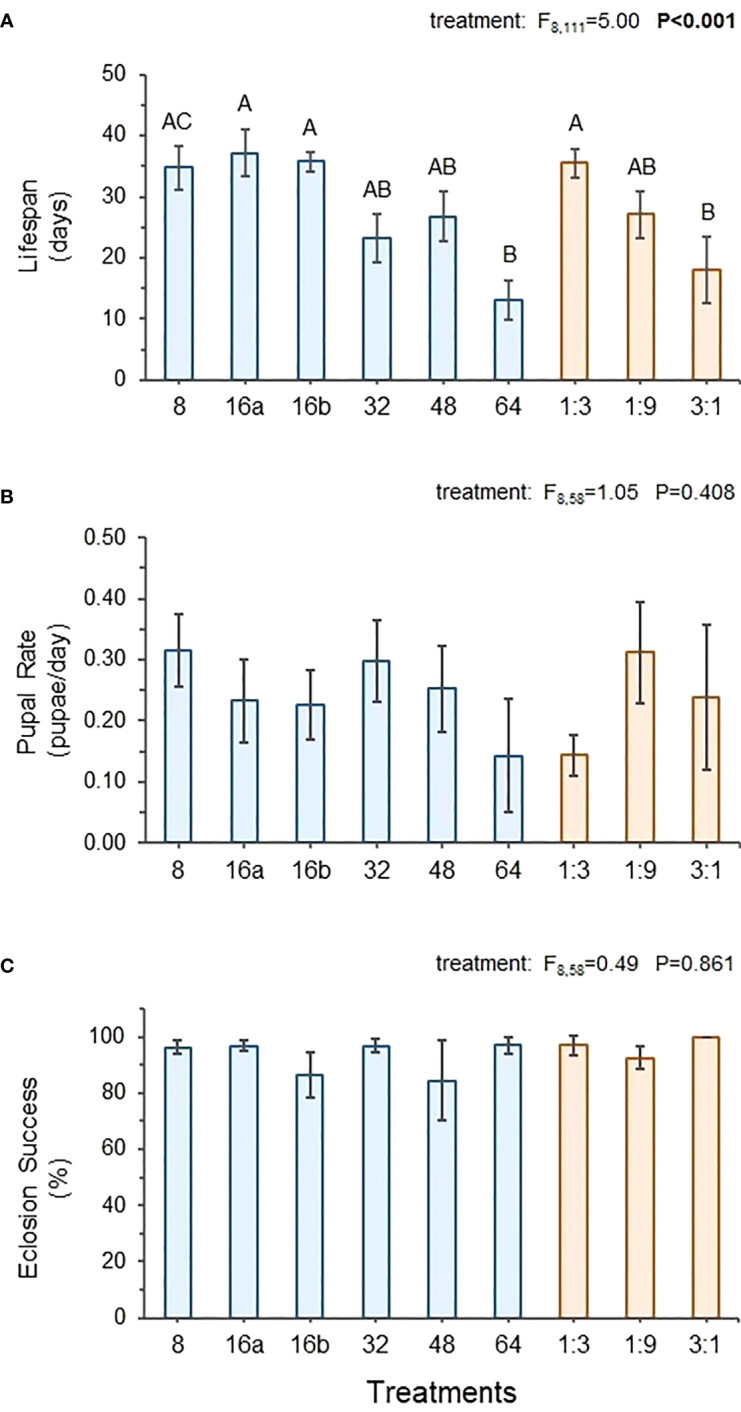
Average lifespan of adult female flies **(A)**, and average pupal rate **(B)** and eclosion success of F1 flies **(C)** from females exposed to different interval and control treatments. ANOVA results for performance variables across treatments are shown. Bolded values indicate statistically significant P-values (P ≤ 0.05). Different letters indicate significant *post-hoc* (Tukey HSD) differences between treatments (P ≤ 0.05).

Feeding interval had a significant impact on fly lifespan but little impact on pupal rate or eclosion success. **Figure 3A** shows that across the control treatments, lifespan was longest on the optimal 1:3 diet and significantly lower on the protein-rich 3:1 diet. Lifespan in the 8-hr and both 16-hr treatments were similarly high, while lifespan in the 32- and 48-hr treatments was somewhat reduced. The 64-hr treatment displayed the shortest lifespan, which was approximately 60% lower than the 8-hr, 16a-, 16b-hr, and 1:3 diets and more similar to the 3:1 control treatment (**Figure 3A**). Mean pupal rate did vary substantially across treatments but displayed too much variability for statistical differences to be detected (**Figure 3B**). Eclosion success was consistently high across both control and interval treatments, showing no statistical differences (**Figure 3C**).

## 4 Discussion

When consumers have access to multiple diets that are individually imbalanced but together complementary, they can feed selectively to achieve a specific macronutrient balance, i.e., intake target. In this study, we explored the temporal aspects of nutrient regulation by controlling the amount of time flies had to regulate and, to a lesser extent, the scheduling of diet switches. We found that both the time interval over which flies had to regulate their macronutrient intake and the timing of diet switching had significant effects on their consumption patterns and performance. Across interval treatments, flies generally consumed larger amounts of diet at the beginning of the experiment than the end, regardless of diet p:c ratio; however, this trend was only statistically significant for the shorter-interval treatments (8-, 16a-, and 16b-hr). Flies in the shorter-interval treatments also consumed significantly more diet overall than the longer-interval or control treatments. Despite differences in consumption patterns, flies in all but one interval treatment reached a statistically-similar intake p:c ratio by the end of the experiment, highlighting the drive to regulate macronutrient intake over long and short time periods. Interestingly, the only treatment to show a different intake ratio was 16a-hr, which had the same regulation time as the 16b-hr treatment (8 hours of access to each diet each day), but a different diet switching schedule.

In the 16a-hr treatment, the diets were switched at 4pm each day and for the 16b-hr treatment they were switched at noon (**Table 1**). The most likely way for switching schedule to impact feeding behavior, would be if it affected the innate diurnal activity patterns of *D. suzukii*. Numerous studies have shown that adult *D. suzukii* are crepuscular and have the highest activity levels at dawn (6am-8am) and dusk (6pm-10pm) (35–37). In both the 16a- and 16b-hr treatments, flies had access to one diet in the morning and the other in the evening, alternating each day, so it seems unlikely that disruptions in diurnal activity alone would explain the regulation towards a more protein-biased intake. Compensatory feeding has been frequently observed in *D. melanogaster* (25, 39) but less so in *D. suzukii*, largely due to the focus on larval, rather than adult, feeding behavior (33, 34, 40). The consumption pattern in the 16b-hr treatment shows evidence of compensatory feeding across days, as the flies alternated their consumption of both diets in an inverse fashion (**Figure 2C**). The more protein-biased intake ratio observed in the 16a-hr treatment, however, was due to consistently higher daily consumption of the 3:1 diet, and an apparent lack of compensatory feeding, at least across days (**Figure 2B**). It is possible that the act of transferring flies later in the evening, as opposed to midday, disrupted or changed their activity patterns in unexpected ways. However, the diets were alternated within and across days, making it unclear how this perturbation would cause a change in macronutrient regulation and/or disrupt compensatory feeding.

In addition to relative consumption, different patterns of total diet consumption were also observed across treatments. Total diet consumption in the 8-, 16a-, and 16b-hr treatments was on average 37.5% higher than the 32-, 48-, and 64-hr treatments and 50% higher than the control treatments. These differences may reflect different strategies used by flies in each treatment to regulate their macronutrient intake. When consumers have multiple resources available simultaneously, they can feed selectively across them to reach their intake target in real time. However, when only imbalanced resources are available, consumers can choose to consume enough of the resource to meet their requirement for one macronutrient, while over- or under-ingesting the other, or they can consume the amount that minimizes both nutritional deficiencies and excesses (17). When different diets are available in a sequence, flies, as already mentioned, may also engage in compensatory feeding, particularly when a preferential resource is offered after they are forced to consume an imbalanced resource for a period of time. In this study, flies did not have the opportunity to regulate their intake of both diets simultaneously but instead over increasingly longer time intervals. As a result, the feeding behavior observed across treatments may represent the combination of multiple strategies used to mitigate imbalanced resources. It is also possible that flies adapted their feeding behavior due to learning. In this way, flies that have access to both diets over a shorter timeframe may not exhibit the same strategy as those with access to only one diet for a longer period.

Flies in the 8-, 16a-, and 16b-hr treatments were exposed to both diets each day, while flies in the 32-, 48-, and 64-hr treatments only had access to one diet per day or couple of days. The increased consumption observed in the shorter-interval treatments could be the result of flies consuming more of both diets in order to reach their intake target on a per-day basis, since they had access to both diets within a 24-hr period. Conversely, those in the longer-interval treatments may have consumed less of the available imbalanced diet as a means of minimizing nutritional deficits/excesses on a daily basis. It is also possible that fly feeding behavior was impacted by learning and/or neophilia, a phenomenon where exposure to new stimuli can have a stimulatory effect on behavior. Studies with insects have shown that feeding behavior is largely driven by the cumulative interaction between neuronal factors that stimulate feeding and those that deter it (41–43). As with compensatory feeding, exposure to low- or high-quality diets, as well as those that are familiar or novel, can have strong impacts on consumption rate and/or feeding time (44–48). Concerning neophilia, since the diets in the shorter-interval treatments were changed more frequently, it is possible that fly consumption was stimulated by the recurrent exposure to new diets, while flies in the longer-interval treatments were habituated to their diet and fed less. This is potentially supported by the fact that total diet consumption fell over time in the 8-, 16a-, and 16b-hr treatments, as well as controls. Of course, this could also be evidence that flies became adapted to their feeding schedules. Identifying learned feeding responses was not possible in this study as feeding behavior was not measured. There is little evidence, however, that the flies in this study had positive or negative learned responses to either diet, as they readily consumed both diets across treatments.

Despite different patterns in total and relative consumption across treatments, the average intake ratios were quite similar across interval treatments. The daily intake ratios, however, fluctuated considerably more in the longer- (32-, 48-, 64-hr) versus the shorter-interval (8-, 16a-, and 16b-hr) treatments due to the experimental conditions, and this had impacts on fly performance. Although pupal rate and eclosion success of F1 flies did not vary significantly across treatments, fly lifespan did. Lifespan was reduced in the longer-interval treatments, suggesting that there are physiological benefits associated with regulating macronutrient intake on shorter timeframes. More specifically, the lifespan data show that being able to regulate within a 24-hr period is preferable to longer periods, as the 8-, 16a-, and 16b-hr treatments showed similar and distinctly longer lifespans than the other treatments where regulation occurred on an interval longer than 24 hours. This is perhaps not surprising, considering that many physiological processes associated with feeding behavior, metabolism, and resource allocation are regulated on a 24-hr circadian cycle (49–53). It is also possible for changes in resource availability and feeding to reset circadian clocks (54, 55). These results show how the temporal aspects of nutrient regulation can impact fly fitness in ways that are comparable to the better-documented effects of diet quality. In fact, the effect of time interval on fly lifespan in this study mirrored the impacts of diet quality in the control treatments.

The performance data showed that flies fed the optimal diet of 1:3 had the longest average lifespan, while those in the protein-biased 3:1 treatment exhibited significantly shorter lifespans. The average lifespans of flies in the 8-, 16a-, and 16b-hr treatments were similar to those in the 1:3 control treatment, while the 32- and 48-hr treatments were similar to the 1:9 control treatment, and the 64-hr treatment to the 3:1 control treatment. One might expect this association to simply be the result of flies in the shorter-interval treatments being able to reach their intake target more quickly, while those in the longer-interval treatments spent more time feeding on the unbalanced diets. Surprisingly, the average intake ratio for all interval treatments were more protein-biased than the expected 1:3 intake target (**Figure 1B**), with only the 32-hr treatment exhibiting a ratio between 1:4 to 1:2. It is unclear why flies would regulate for a more protein-biased ratio. The intake target for *D. suzukii* has largely been based on choice tests done with larvae rather than adults, so it is possible that adults have higher protein requirements, particularly female flies that need to produce protein-rich eggs. It is also possible that flies consume protein-biased resources to a greater extent when it is available, given that dietary protein is more likely to be limiting than carbohydrates. It should also be noted that multiple nutritional optima can exist depending on the physiological demands experienced by the consumer. For instance, Lee et al. (11) found that female *D. melanogaster* adults prioritized maximal egg production when allowed to regulate their macronutrient intake and selected a more protein-biased diet, but he also found that maximal lifespan occurred at a more carbohydrate-biased ratio. Given that we observed differences in average lifespan but no differences in pupal rate across treatments in this study, the flies were likely prioritizing reproduction at the cost of longevity, as was observed in Lee et al. (11).

In conclusion, the results of this study suggest that the temporal aspects of nutrient regulation have important implications for consumer fitness that can be as significant as diet quality alone. It is notable that despite facing greater daily variability in diet p:c, the flies in the longer-interval treatments were able to regulate their macronutrient intake as precisely as those in the shorter-interval treatments. This suggests that the physiological mechanisms that underlie nutrient regulation operate effectively over short and long timeframes. It is also interesting to note that the way the flies regulated their diet-specific consumption to meet their intake target varied across feeding intervals, with intake of both carbohydrate- and protein-biased diets varying significantly across treatments. These conclusions are relevant for understanding the temporally variables associated with nutritional environments in natural systems. For many animals, including insect herbivores, nutrient availability can vary in predictable or stochastic ways. For instance, plant macronutrient profiles show strong temporal variability at the scale of individual plants as tissues age and environmental changes (26, 56), but they can also vary generationally as seasons change (14). Temporal variability in nutrient dynamics represent an important constraint to individual consumers attempting to regulate their intake but also populations over time (14). As global climate change promises to alter current ecological patterns (57–60), consumers will likely have to deal with qualitative and temporal changes in their resource base. These interactions may be particularly important for *D. suzukii*, as their resource base consists of numerous host plants with complex microbial associations (19, 30). Further research to characterize the role that time plays in nutrient regulation will help researchers better understand the mechanisms available to consumers for adaptation and will allow more accurate predictions about the repercussions of environmental changes.

## Data availability statement

The raw data supporting the conclusions of this article will be made available by the authors, without undue reservation.

## Author contributions

CD is responsible for the writing of this article and the creation of the figures. WH is responsible for the editing of the manuscript. CD is responsible for the experimental design and CD and WH for the generation of the concepts and ideas provided. All authors contributed to the article and approved the submitted version.
